# Supplementary material for: Genome-Wide Identification of GmSPS Gene Family in Soybean and Expression Analysis in Response to Cold Stress
Source: Int J Mol Sci. 2023 Aug 17;24(16):12878. doi: 10.3390/ijms241612878 (PMC10454306; doi:10.3390/ijms241612878)
Supplement: Supplementary file 1 [file ijms-24-12878-s001.zip › Figure S1.pdf]

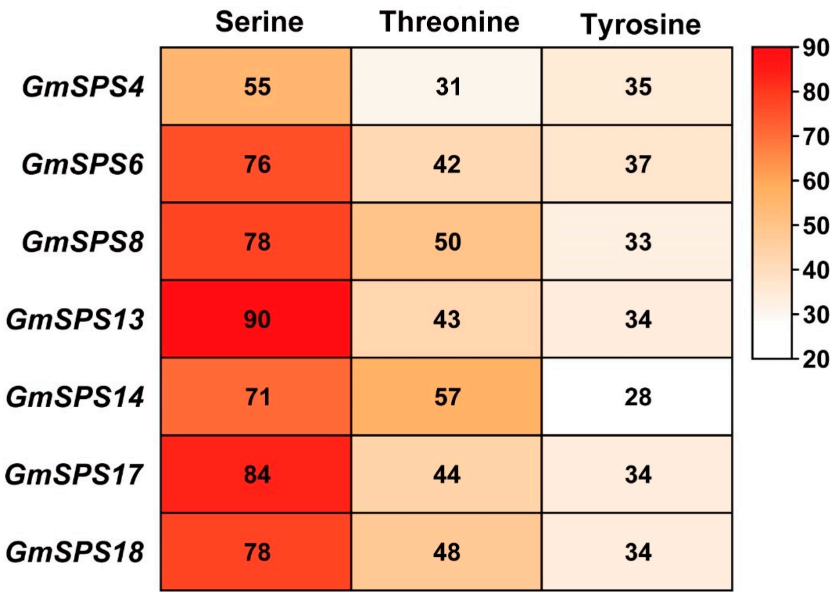

Figure S1. Phosphorylation site analysis of GmSPS proteins. The numbers in the first, second and third columns represent the number of serine, threonine and tyrosine in the corresponding protein sequence, respectively. The color bar represents number scale.
